# Comparison of perioperative outcomes in frail patients following multilevel lumbar fusion surgery with and without the implementation of the enhanced recovery after surgery protocol

**DOI:** 10.3389/fsurg.2022.997657

**Published:** 2022-11-02

**Authors:** Peng Cui, Shuaikang Wang, Peng Wang, Lijuan Yang, Chao Kong, Shibao Lu

**Affiliations:** ^1^ Department of Orthopedics, Xuanwu Hospital Capital Medical University, Beijing, China; ^2^ National Clinical Research Center for Geriatric Diseases, Beijing, China; ^3^ Department of Pathology, West China Hospital, Sichuan University, Sichuan, China

**Keywords:** enhanced recovery after surgery, frail, multilevel, lumbar fusion surgery, propensity score matching

## Abstract

**Background:**

Enhanced recovery after surgery (ERAS) is an evidence-based multimodal perioperative management designed to reduce the length of stay (LOS) and complications. The purpose of the present study is to evaluate the recovery of physiological function, LOS, complications, pain score, and clinical efficacy in frail elderly patients undergoing multisegment fusion surgery after the implementation of the ERAS protocol.

**Methods:**

Frail patients older than 75 years undergoing multilevel lumbar fusion surgery for degenerative discogenic conditions, lumbar spinal stenosis, and lumbar spondylolisthesis from January 2017 to December 2018 (non-ERAS frail group) and from January 2020 to December 2021 (ERAS frail group) were enrolled in the present study. Propensity score matching for age, sex, body mass index, and smoking status was performed to keep comparable characteristics between the two groups. Further recovery of physiological function, LOS, complications, pain score, and clinical efficacy were compared between the groups.

**Results:**

There were 64 pairs of well-balanced patients, and the clinical baseline data were comparable between the two groups. There was significant improvement in terms of recovery of physiological function (10.65 ± 3.51 days vs. 8.31 ± 3.98 days, *p* = 0.011) and LOS (12.18 ± 4.69 days vs. 10.44 ± 4.60 days, *p* = 0.035), while no statistical discrepancy was observed with regard to complications between the groups, which indicated favorable outcomes after the implementation of the ERAS protocol. Further analysis indicated that more patients were meeting a minimally clinical important difference for the visual analog score for the legs and the Oswestry Disability Index in the ERAS frail group. With regard to postoperative pain, the score was higher in the ERAS frail group than in the non-ERAS frail group on postoperative day (POD) 1 (4.88 ± 1.90 in the ERAS frail group vs. 4.27 ± 1.42 in the non-ERAS frail group, *p* = 0.042), while there was no significant discrepancy on POD 2 (3.77 ± 0.88 in the ERAS frail group vs. 3.64 ± 1.07 in the non-ERAS frail group, *p* = 0.470) and POD 3 (3.83 ± 1.89 in the ERAS frail group vs. 3.47 ± 1.75 in the non-ERAS frail group, *p* = 0.266).

**Conclusions:**

In this retrospective cohort study, we found a significant improvement in terms of LOS, recovery of physiological function, and clinical efficacy after the implementation of the ERAS protocol in elderly and frail patients undergoing multilevel lumbar fusion surgery, while there was no significant discrepancy with regard to complications, 90-day readmission, and postoperative pain.

## Introduction

Enhanced recovery after surgery (ERAS) is an evidence-based, multidisciplinary perioperative approach adopted to decrease postoperative adverse events by mitigating stress response in patients following surgical intervention (1–4). First introduced for colon surgery, the ERAS protocol has been implemented successfully in various surgical specialties. Substantial attention has been paid to spine surgery, and several studies have found that patients undergoing lumbar fusion surgery (short-segment or multilevel) can benefit from the implementation of the ERAS protocol (5–10). Studies have demonstrated that ERAS for lumbar fusion could reduce hospitalization costs, postoperative pain, and complications, while facilitating the recovery of physiological function without adversely affecting readmission rates; this is the case irrespective of whether the protocol is implemented preoperatively, intraoperatively, or postoperatively (11, 12).

There is now an increased focus on desirable perioperative outcomes in vulnerable patients due to the increasing incidence of age-related disorders. Frailty is clinically defined as a syndrome characterized by decreased physiological reserve that can predispose patients undergoing surgery to suboptimal outcomes (13, 14). Moreover, previous studies have shown that frail patients are susceptible to an increased risk of complications, a longer length of stay (LOS), and more hospitalization expenditures arising from lumbar surgery (15, 16). Accurate risk stratification and predicting postoperative complications in time are imperative in older patients undergoing lumbar fusion surgery. The Fried frailty phenotype was described by Fried and colleagues (17), which is comprised of five variables, namely unintentional weight loss, self-reported exhaustion, low physical activity, slowness, and weakness. The score assigns one point for any of these factors and is calculated by adding each variable.

Previous studies have demonstrated the clinical efficacy of the ERAS protocol in lumbar fusion surgery; however, there has been a lack of sufficient data pool for evaluating ERAS in frail patients following lumbar fusion surgery, especially multisegment lumbar fusion surgery (8). Furthermore, with increasing age, elderly patients often suffer from comorbidities, making the vulnerable among them more prone to an increased risk of suboptimal outcomes (18). Against this background, this study aims to evaluate the return of physiological function, LOS, complication rates, pain scores, and clinical efficacy in frail elderly patients undergoing multisegment fusion surgery after the implementation of the ERAS protocol.

## Methods

### Population

This was a retrospective cohort study. This study was approved by the institutional review board in Xuanwu Hospital Capital Medical University (No. 2018086). Informed consent was waived due to the nature of the study design. Consecutive patients who underwent multilevel lumbar fusion surgery, defined as fusion segments greater than or equal to 3 before and after the implementing the ERAS protocol, were reviewed in this study. Inclusion criteria were (1) age >75 years; (2) undergoing multilevel lumbar fusion surgery for degenerative discogenic conditions, lumbar spinal stenosis, and lumbar spondylolisthesis; and (3) completed preoperative data. A multidisciplinary appraisal team was established in 2019 at our institution with the aim of minimizing selection bias, and the Fried phenotype score was evaluated by specially trained nurses. A patient was defined as frail if the score was >2. Exclusion criteria were (1) history of spinal surgery; (2) concomitant cervical surgery or thoracic spine surgery; and (3) lack of clinical data. The ERAS protocol was implemented in July 2019 to increase the reliability and comparability of the data, patients reviewed from January 2017 to December 2018 were classified as non-ERAS frail group, and those reviewed from January 2020 to December 2021 were classified as ERAS frail group. Propensity score matching for age, sex, body mass index (BMI), and smoking status was performed to maintain comparable clinical characteristics.

### Enhanced recovery after surgery interventions

The ERAS protocol for multilevel lumbar fusion surgery was fully implemented in our department in July 2019 and a multidisciplinary assessment team was established. The ERAS program is a patient-specific perioperative management approach, and a tailor-made management regimen is adopted for patients by following ERAS principles. Our ERAS protocol consisted of preoperative, intraoperative, and postoperative interventions. The perioperative measures were (1) perioperative education and counseling: informing the patients about the risk of surgery and describing the ERAS pathway to ensure their understanding; (2) nutritional assessment: patients with malnutrition were provided with personalized and diet guidance and nutritional supplements from an expert nutritionist before surgery; (3) cessation of smoking and alcohol: 2 weeks before surgery; (4) no prolonged fasting: eating was permitted up to 6 h prior to surgery and carbohydrate-containing drinks were allowed up to 2 h before surgery; (5) multimodal analgesia: various analgesics were used according to pain stratifications; (6) antibiotic prophylaxis: within 1 h of the incision. Intraoperative interventions were (1) tranexamic acid: within half an hour of incision; (2) maintenance of normothermia: keeping temperature at 36–37°C; (3) local infiltration analgesia: 10 ml ropivacaine and 10 ml lidocaine; (4) standard anesthetic protocol: total intravenous anesthesia-based anesthetic technique with propofol, lidocaine, ketamine, ketorolac, antiemetics, and with up to 0.5% minimum alveolar concentration–inhaled anesthetics. Postoperative interventions were (1) early oral feeding: oral feeding after recovery from anesthesia; (2) early ambulation: patients with multilevel lumbar fusion surgery were suggested to ambulate out-of-bed with or without assistance within 48 h after surgery; (3) early removal of the bladder catheter: consider removing the catheter after 24 h; (4) multimodal analgesia: similar to the preoperative multimodal analgesia regimen with a patient-controlled analgesia pump.

### Collected variables

Patient-specific and procedure-specific variables were reviewed from the medical records. The patient-specific perioperative variables included age, sex, BMI, smoking status, visual analog score (VAS) for the back and legs, Oswestry Disability Index (ODI) score before and after surgery, American Society of Anesthesiologists (ASA) classification, and Charlson comorbidity index (CCI) (19). The procedure-specific variables included operation time, intraoperative blood loss, intraoperative blood transfusion, LOS, fusion segments, 90-day readmission, and postoperative complications (i.e., deep vein thrombosis, pneumonia, surgical site infection, bacteremia, uroschesis, urinary tract infection, myocardial ischemia, neurological deficit, hematoma, delirium, spinal fluid leakage, and nausea and vomiting). We recorded the time to first ambulation, time to first bowel movement, and time to void, and the return of physiological function was defined as the sum of these parameters. Clinical efficacy was compared between the two groups according to the minimal clinically important difference (MCID) with a cutoff of 12.8 points for the ODI, 1.2 points for back pain, and 1.6 points for leg pain (20).

### Surgical technique

A standard midline approach was performed to expose the posterior elements. The nerve roots were decompressed by hemilaminectomy or laminectomy according to the preoperative lumbar symptoms, radicular symptoms, and MRI. Spinal instrumentation was performed using a pedicle screw-rod construct, followed by a decompression of responsible segments with transforaminal lumbar interbody fusion. All surgeries were performed by the same team.

### Statistical methods

Continuous variables were summarized as mean ± standard deviation when data were normally distributed, while categorical variables were expressed as frequencies and percentages. The continuous variables were analyzed using independent two-sample *t*-tests and categorical variables were compared using a chi-square test or the Fisher’s exact test. All statistical analyses were performed using SPSS software version 25.0 (SPSS, Inc., Armonk, NY, USA). *P*-values < 0.05 were considered statistically significant.

## Results

### Demographics

The detailed demographic patient data are presented in Table 1. After propensity score matching for age, sex, BMI, and smoking status, there were 64 pairs of well-balanced patients. The mean age was 79.94 ± 3.23 years and BMI was 25.24 ±^ ^2.98 kg/m^2^ with 73.44% of women in the ERAS frail group. Analogously, the mean age was 79.32 ± 3.21 years and BMI was 25.69 ± 2.56 kg/m^2^ with 75.00% of women in the non-ERAS frail group. Patient-specific and procedure-specific baseline characteristics were comparable in both cohorts. CCI, ASA, pre-ODI, and pre-VAS for the back and legs were similar. In addition, there were no significant differences in terms of fusion segment, operation time, estimated blood loss, or intraoperative blood transfusions.

**Table 1:** Patients’ demographics.

Variable	non-ERAS frail	ERAS frail	*P*
Sample size	64	64	
Age	79.32 ± 3.21	79.94 ± 3.23	0.281
Female	48/64	47/64	0.840
BMI	25.69 ± 2.56	25.24 ± 2.98	0.355
CCI	1.91 ± 1.65	2.05 ± 1.85	0.652
Smoking	4/64	6/64	0.510
Fusion segment			0.829
3	41	40	
4	18	17	
5	5	7	
Pre-ODI	48.15 ± 9.78	48.55 ± 13.98	0.880
Pre-VAS for the back	4.50 ± 2.18	4.31 ± 2.36	0.758
Pre-VAS for the legs	4.96 ± 1.56	4.59 ± 2.01	0.407
ASA			0.529
I	0	1	
II	12	16	
III	51	46	
IV	1	1	
Operation time	279.03 ± 63.35	265.50 ± 62.61	0.225
EBL	597.85 ± 375.32	604.22 ± 333.92	0.919
Intraoperative blood transfusion	569.48 ± 559.74	559.00 ± 456.65	0.908

BMI, body mass index; CCI, Charlson comorbidity index; ODI, Oswestry Disability Index; VAS, visual analog score; ASA, American Society of Anesthesiologists classification; EBL, estimated blood loss.

### Perioperative outcomes

Perioperative characteristics are given in Table 2. There was no significant difference with regard to complication rates, 90-day readmission, post-ODI, post-VAS for the back, and post-VAS for the legs for both cohorts. However, there was a significant reduction in the LOS in the ERAS frail group (12.18 ± 4.69 days vs. 10.44 ± 4.60 days, *p* = 0.035).

**Table 2:** Perioperative outcomes between the enhanced recovery after surgery frail group and the non-enhanced recovery after surgery frail group.

Variable	non-ERAS frail (*n* = 64)	ERAS frail (*n* = 64)	*P*
Complications			
Deep vein thrombosis	0 (0)	1 (1.56%)	1
Pneumonia	1 (1.56%)	3 (4.69%)	0.611
Surgical site infection	3 (4.69%)	4 (6.25%)	1
Bacteremia	1 (1.56%)	0 (0)	1
Uroschesis	4 (6.25%)	3 (4.69%)	1
Urinary tract infection	4 (6.25%)	3 (4.69%)	1
Myocardial ischemia	3 (4.69%)	3 (4.69%)	1
Neurological deficit	0 (0)	1 (1.56%)	1
Hematoma	2 (3.13%)	1 (1.56%)	1
Delirium	0 (0)	1 (1.56%)	1
Spinal fluid leakage	1 (1.56%)	0 (0)	1
Nausea and vomiting	6 (9.38%)	3 (4.69%)	0.489
Complication rates	18 (28.13%)	15 (23.44%)	0.544
LOS	12.18 ± 4.69	10.44 ± 4.60	0.035
90-day readmission	7 (10.94%)	5 (7.81%)	0.544
Post-ODI	33.38 ± 23.89	33.09 ± 24.00	0.945
Post-VAS for the back	2.92 ± 1.60	2.91 ± 1.72	0.954
Post-VAS for the legs	2.95 ± 1.81	2.78 ± 1.71	0.588
Return of physiological function			
1st ambulation POD	3.42 ± 1.72	2.38 ± 1.72	0.010
1st void POD	2.92 ± 1.79	2.50 ± 1.84	0.322
1st bowel movement POD	4.31 ± 1.32	3.44 ± 1.47	0.010

LOS, length of stay; ODI, Oswestry Disability Index; VAS, visual analog score; POD, postoperative day.

**Table 3:** Clinical efficacy described by recovery for the Oswestry Disability Index and visual analog score according to the minimal clinically important difference between groups.

Achieved MCID for	Non-ERAS frail (*n* = 64)	ERAS frail (*n* = 64)	*P*
ODI	40 (62.5%)	51 (79.69%)	0.032
VAS for the back	39 (60.94%)	43 (67.19%)	0.461
VAS for the legs	34 (53.13%)	45 (70.31%)	0.045

ODI, Oswestry Disability Index; VAS, visual analog score; MCID, minimal clinically important difference.

### Recovery of physiological function

**Figure 1:**
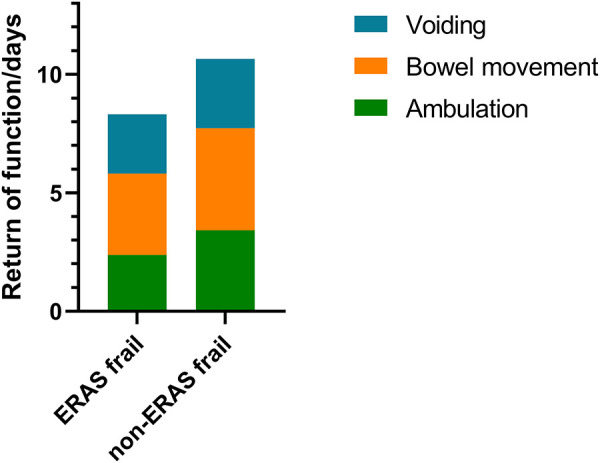
Stacked bar graph denoting recovery of physiological function for the ERAS frail group and the non-ERAS frail group. ERAS, enhanced recovery after surgery.

Significant improvements were seen on the first day of ambulation (3.42 ± 1.72 days vs. 2.38 ± 1.72 days, *p* = 0.010) and the first day of bowel movement (4.31 ± 1.32 days vs. 3.44 ± 1.47 days, *p* = 0.010) in the ERAS frail group. On average, the first day of bladder voiding occurred 0.42 days earlier (2.92 ± 1.79 days vs. 2.50 ± 1.84 days, *p* = 0.322) in the ERAS frail group, although no significant difference was observed. There was significant improvement in terms of recovery of physiological function in the ERAS frail group (10.65 ± 3.51 days vs. 8.31 ± 3.98 days, *p* = 0.011). The detailed characteristics are displayed in Figure 1.

### Postoperative pain

**Figure 2:**
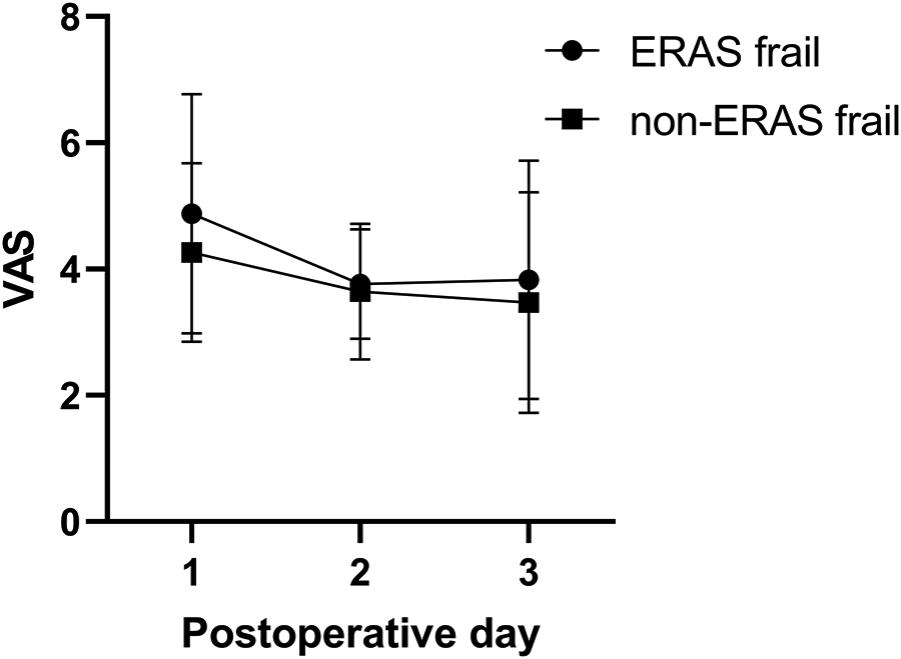
Pain scores on POD 1–3 between the ERAS frail group and the non-ERAS group. POD, postoperative day; ERAS, enhanced recovery after surgery.

The mean pain scores on postoperative days (PODs) 1–3 between the cohorts are illustrated in Figure 2. A significant difference was observed in the pain score on POD 1 (4.88 ± 1.90 in the ERAS frail group vs. 4.27 ± 1.42 in the non-ERAS frail group, *p* = 0.042), while there was no significant difference on POD 2 (3.77 ± 0.88 in the ERAS frail group vs. 3.64 ± 1.07 in the non-ERAS frail group, *p* = 0.470) and POD 3 (3.83 ± 1.89 in the ERAS frail group vs. 3.47 ± 1.75 in the non-ERAS frail group, *p* = 0.266).

### Clinical efficacy

There were 51 (79.69%) patients in the ERAS group and 40 (62.50%) patients in the non-ERAS frail group meeting an MCID for the ODI, respectively (*p* = 0.032). In addition, there was substantial improvement in the VAS for the legs in the ERAS frail group compared with that in the non-ERAS frail group (70.31% vs. 53.13%, *p* = 0.045). More patients were meeting an MCID for the VAS for the back in the ERAS frail group, without a significant discrepancy (67.19% vs. 60.94%, *p* = 0.461).

## Discussion

ERAS is an evidence-based multidisciplinary perioperative pathway designed to achieve early convalescence, a reduction of LOS, and postoperative complications (5, 21, 22). Conspicuous perioperative outcomes in previous studies resulted in ERAS gaining in popularity in spine surgery. Although ERAS studies have increased exponentially, there is a dearth of studies investigating the implementation of the ERAS protocol in frail older patients (>75 years) (8). The present study indicated that the implementation of the protocol amplified the recovery of physiological function, improvement of clinical efficacy, and reduction of LOS.

Frailty is clinically defined as a syndrome characterized by a decreased physiological reserve, predisposing patients to undergo surgery to avoid suboptimal outcomes. Further, multilevel lumbar fusion surgery exhibits higher complication rates and a longer LOS than their short-level counterparts (18). Therefore, if there are no external meticulous interventions, frail elderly patients would incur an increased risk of suboptimal postoperative outcomes. In a recently published retrospective study of frail patients following 1- or 2-level transforaminal lumbar interbody fusion, Porche et al. (8) indicated that ERAS significantly improved the LOS compared with their non-frail counterparts. In the present study, we found a significant reduction in the LOS in the ERAS frail group, though there was no significant difference in complications. Based on clinical experience, postoperative wound pain is correlated with patient satisfaction (23). Hence, a multimodal analgesia regimen as a part of an ERAS protocol should help maintain pain in the tolerable range. In this study, there was a significant difference in the pain score on POD 1, while there was no significant discrepancy on POD 2 and POD 3. The pain score appeared to have decreased from POD 1 to POD 3, especially in the non-ERAS frail group. In our previously published study (24), we stated that the patient-controlled multimodal analgesia pump is usually removed on POD 3 in our department, and this practice might account for the pain score being a little higher on POD 3 than on POD 2 in the ERAS frail group.

Early ambulation is the backbone of the ERAS protocol, and ERAS is designed to reduce adverse events based on a theoretical rationale for diminishing surgical-related stress response and insulin resistance (25). Hence, early recovery of physiological function occurs after implementing the ERAS protocol theoretically. Consistent with Proche et al., the total days for recovery of physiological function were significantly lower in the ERAS frail group (pre-ERAS: 6.7 days, post-ERAS: 3.4 days, *p* < 0.001). In this study, the first day of ambulation occurred on average 1.04 days earlier, the first day of bowel movement occurred on average 0.87 days earlier, and the first day of bladder voiding occurred 0.42 days earlier in the ERAS frail group than in the non-ERAS frail group, respectively. Furthermore, the number of days to the recovery of physiological function was significantly less, with an average of 2.34 days earlier in the ERAS frail group.

Clinical efficacy is evaluated according to patient-reported health-related quality-of-life questionnaires including the ODI and VAS in spinal surgery studies. However, even subtle changes can yield statistically significant differences in sample sizes and measurement accuracy, and these are sufficient. Therefore, the MCID suggests a threshold to assess clinical efficacy, which makes intergroup analysis intuitive and explicit (20). In a retrospective study, Ayling et al. (26) indicated that there was no significant difference in the ODI and numeric rating scale between patients undergoing 1- to 2-level open transforaminal lumbar interbody fusion or minimally invasive transforaminal lumbar interbody fusion. Further analysis suggested that a higher baseline leg pain score predicted achieving the MCID in both cohorts. Jacob et al. (23) conducted a retrospective study suggesting that meeting an MCID for the back and leg pain was associated with patient satisfaction in lumbar decompression patients. Our study showed a significant improvement in the ODI and VAS after the performance of the procedures both in the ERAS frail group and in the non-ERAS frail group. In addition, despite finding the analogous preoperative and postoperative ODI, VAS for the back, and VAS for the legs, we found a significantly increased number of patients who met an MCID for the ODI and VAS for the legs after the implementation of the ERAS protocol. More patients in the ERAS frail group met an MCID in the VAS for the back; however, there was no significant difference because the postoperative patient-reported outcomes included in this study were evaluated before discharge, which provides favorable evidence for immediate recovery for daily activities after implementing the ERAS protocol in clinical practice.

This study was not without limitations. First, the study suffered from inherent limitations associated with retrospective analysis. Second, we did not perform multivariate analysis for patients who did not meet an MCID on the grounds of insufficient statistical power due to the small sample size. Finally, long-term patient-reported outcomes were not included in this study, as this study primarily focused on ERAS exposure with frailty as the variable, whereas ERAS is a multimodal management approach focusing on perioperative outcomes. Further multicenter studies with large cohorts are required to confirm our findings.

## Conclusion

In this retrospective cohort study, we found a significant improvement in terms of the LOS, recovery of physiological function, and clinical efficacy after the implementation of the ERAS protocol in elderly and frail patients undergoing multilevel lumbar fusion surgery, while there was no significant discrepancy with regard to complications, 90-day readmission rates, and postoperative pain.

## Data Availability

The original contributions presented in the study are included in the article/Supplementary Material; further inquiries can be directed to the corresponding author/s.
